# Book Reviews

**Published:** 1832-03

**Authors:** 


### CRITICAL ANALYSES.

Quæ laudanda forent, et quæ culpanda, vicissim,

Illa prius, cretâ; mox hæc, carbone, *notamus*.—PERSIUS.

*A Practical Compendium of Midwifery; being the Course of Lectures on Midwifery, and on the Diseases of Women and Infants, delivered at St. Bartholomew's Hospital, by the late* ROBERT GOOCH, M.D. Prepared for publication by GEORGE SKINNER, Member of the Royal College of Surgeons, London. —12mo. pp. 358. Longman and Co. London, 1831.

WE have read this volume attentively, and have no doubt that Mr. SKINNER gives a faithful transcript of Dr. GOOCH's lectures as they were delivered some years ago; but it is very certain that, if Dr. Gooch had, in the latter part of his life, undertaken to write a "Compendium of Midwifery," he would have left a performance much more creditable to his own reputation, and more satisfactory and instructive to his readers. It is not fair, indeed, to compare the work before us with other written works: it should be compared with other courses of lectures, and, forming an estimate of its merits in this way, we feel justified in saying that it conveys as much knowledge in its department as any other lectures with which we are acquainted. It is not our intention to enter into an analysis of each lecture; but, for the purpose of giving our readers some idea of the manner in which the various subjects are discussed, we shall select such extracts as appear to us the most practically useful.

In treating upon *Chlorosis*, Dr. Gooch recommends a trial of electricity, in case menstruation does not take place when the general health is restored by other means. We refer to this passage in his lecture, because we believe that electricity is rarely, if ever, tried in such cases by the generality of practitioners. Although the patient does not menstruate, she usually experiences pains in the loins, headach, shooting pains in the breasts, &c., at the customary intervals of menstruation: "profiting by an opportunity of this kind, we must pass slight electric shocks through the pelvis; this has succeeded on the second day of its use, and the female has felt the blood issuing from the pudenda even before she had time to quit the room."

*Amenorrhœa.* A woman may have passed over that period of life when chlorosis is likely to occur, and menstruation may have gone on regularly for a long period, when, from certain causes, its return at the usual period may be prevented, or the discharge may be suddenly checked when it has actually taken place. An interruption of either kind may, in general, be attributed either to mental emotion or to the application of cold.

"We are continually witnessing effects of these causes on the functions of the sexual organs. Sudden terror, or any other passion, will not only check the secretion of milk, but will alter its quality; for if a child sucks under such circumstances, it will be griped. It is an ascertained fact among the assbreeders, that if the foal dies, and another is invested in its skin, and introduced to the mother as her own progeny, the milk will be freely secreted so long as the deception continues; but on her discovering the cheat, the secretion of milk immediately ceases. This proves that mental agitation has a powerful influence over the function of one part of the sexual system, and it will also affect not less powerfully the function of the uterus. I was prescribing for a French lady labouring under a suppression of the menses, and on questioning her, found that she had not menstruated since the Cossacks entered Paris; and it was a well-authenticated circumstance that, at the time the allied armies entered Paris, from a similar mental emotion, a great number of French ladies were thus affected. The French journals of the day contained long lists of patients labouring under this disease. The effect of cold on the uterus during menstruation, or about the time of its occurrence, is a matter of familiar experience; and it is to this influence that the suppression of the menses is, perhaps, the most frequently to be imputed.

"How do these causes operate? On some occasions they seem to excite an inflammatory action in the uterus, and they act on others by producing spasm; differences dependent upon the idiosyncrasies of different individuals. If a young unmarried woman of full habit, with a red face, fleshy, and robust, be exposed to these causes, the consequent cessation of the discharge is followed by pains in the head and back, a sense of weight, pain, and tension about the region of the uterus, which is also tender on pressure, together with a hot skin and rapid pulse. Here the attack seems to be of an inflammatory nature. But when females of a delicate habit, having an irritable nervous system, are subjected to a similar influence, the consequences are irritation and spasm of the uterus, rather than inflammatory disease; for the pain is not continued, the uterus is not tender on pressure, the pulse is neither rapid nor full, and the skin is not hot: on the contrary, there is a general sense of chilliness, and the feet are particularly cold.

"If proper remedies are employed, the disease will in general be cured; but this success is by no means invariable. A young female, fleshy and full of blood, got very wet during menstruation; the discharge was suppressed, to which succeeded headach, a hot skin, and rapid pulse, pain and tenderness in the region of the uterus, &c.: physicians were consulted, but she died; and on examination the uterus was found in a state of mortification, the result of intense inflammation.

"The treatment of this disease must be adapted to the variety of it under which a patient suffers. If of the inflammatory kind, which would be denoted by the presence of febrile symptoms, together with continued pain and tenderness in the region of the uterus, it must be treated by such bleedings, both general and local, as by leeches, as the violence of the symptoms may demand, and by all those other means comprised in the antiphlogistic plan. By such treatment the inflammatory action will most probably be subdued; and on its subsidence, the menstrual discharge will be renewed and proceed in the customary manner.

"But supposing a suppression of this kind occurs in one of the other class of females; here we shall obtain more benefit from that which is termed the antispasmodic, than from the antiphlogistic plan; but in this case it is proper to purge, and that smartly. Put the patient into the warm hip bath, administer diluent drinks, and give antispasmodics, such as five grains of pulv. ipecac. comp. and three grains of camphor, every four hours, until all the symptoms subside. The injection of antispasmodic remedies, such as laudanum or assafœtida, into the rectum, will sometimes act like a charm; in the latter I have great faith: let two drachms of assafœtida be rubbed down with the yolk of an egg, and mixed with six ounces of warm water; this mixture should be gradually injected up the rectum, which being done, a napkin should be closely applied to the part, and every means made use of in order to secure its retention. If you use laudanum, let the vehicle be small in quantity: an injection of this kind may consist of fifty or sixty drops of laudanum in three ounces of water. It is a great error to suppose the benefit is to be in proportion to the quantity of fluid injected: this may be true to some extent of purging injections, but the quantity of those of the anodyne description should be small, both because the active ingredient is less diluted, and the injection is the more likely to be retained. The spasmodic affection having yielded, and the nervous irritation being allayed, menstruation generally ensues. But it may so happen, that when the state of spasm has ceased in one variety of the disease, and that of inflammation in the other, the menstrual discharge may still not supervene. Its recurrence may then be expected at the next period; but if it should be suspended month after month, and year after year, this state is distinguished as that of obstructed menstruation." (P. 14.)

In cases of painful menstruation, Dr. Gooch speaks very confidently of the good effects of camphor: it seems, he says, to act specifically on the uterus, affording considerable relief; but it should not be given when the disease is of the inflammatory kind, in which it may be prejudicial. Dr. Dewees considers the volatile tincture of guaiacum no less a specific for dysmenorrhœa than for the cure of obstructed menstruation. We have given this remedy several times in cases of the former disease, with the greatest advantage; and in one instance the young lady was entirely relieved from her sufferings at the menstrual periods, although she had been afflicted for years, at those times, with very severe pain, which had resisted the use of warm baths, opium, and other common remedies.

*Menorrhagia*. Dr. Gooch describes three kinds of this complaint: the first is characterised by inflammatory symptoms; the second by general debility, and perhaps a relaxed and passive state of the vessels of the uterus; and the third by spasm and irritation.

"These differences must be borne in mind, for we are constantly liable to meet with them in practice. Thus, a woman may menstruate at the regular periods, and the discharge may continue the usual time, yet it may flow with such rapidity that its amount may be more than double that which is customary. Or the discharge may occur at regular periods, and instead of continuing three or four days, as is natural, it may continue ten, twelve, or fourteen days; and as, in the former case, debility is produced by the rapidity of the discharge, in this, the same state is induced by its long continuance. Or, instead of being regular in its periods, it may recur as often as every two or three weeks; and thus also the body is drained of its blood, and the system is debilitated.

"If the disease be of the active and inflammatory kind, we must keep the patient in a recumbent posture on a sofa or mattress, prescribe a low, unirritating diet, together with such saline purgatives as will procure three or four evacuations daily. If there is a full, hard pulse, with a plethoric state of the system, treat it as you would a case of inflammation of any other organ, by copious venesection, and give such medicines as are likely to lower the circulation, as ten grains of the nitrate of potash with ten drops of the tincture of digitalis every six hours. Thus, by a recumbent posture, an unstimulating diet, saline laxatives, bleeding, nitre, and digitalis, the inflammatory symptoms will be subdued, the hot skin will become cool, the pain and tenderness of the uterus will subside, and, as the system becomes tranquillised, the hemorrhage will cease. But this antiphlogistic treatment will not succeed in the spasmodic or irritative form of the complaint. It is difficult at the bedside always to distinguish these two forms; and if, on trial of the antiphlogistic means, no abatement of the symptoms take place, we must have recourse to the antispasmodic remedies, which, after the failure of the former, will generally effect a cure. A lady labouring under this disease, which was thought to be of the acute kind, went through the whole routine of antiphlogistic remedies, without any benefit. I then gave her one grain of ipecacuanha every hour: in eight hours she became nauseated and sick, and the discharge immediately ceased. I had only to keep up this state of nausea for a day or two, and the discharge did not recur. When you have a case of menorrhagia, attended with a quick and irritable pulse, the pain subsiding and recurring, you may be certain that it arises from spasm or irritation, and that it will be relieved by antispasmodic remedies: the two best are, ipecacuanha taken into the stomach, and assafœtida with opium injected into the rectum. A grain of ipecacuanha is to be given every hour till nausea is produced; which state must be maintained for a day or two, by repeating the same dose, as frequently as may suffice for this purpose; and quiet local irritation in the uterus, by injections of assafœtida or opium, as directed in dysmenorrhœa. There is a very marked connexion between the pain and the discharge; for if you can relieve the one, the other will cease." (P. 25.)

In that form of menorrhagia which may be considered as passive, Dr. Gooch speaks highly of the good effect of injections of cold water thrown into the rectum, but he advises us to be cautious in the use of this remedy, if the weather is very cold; first letting the water be used tepid, and gradually quite cold.

In the spasmodic and irritable kinds of menorrhagia, this treatment is not recommended.

*Leucorrhœa.* "The treatment of leucorrhœa is to a great extent empirical. Cold astringents, among the rational practitioners, are in the most general use; but tepid ones are often equally beneficial. Practitioners have exhausted all the cold astringent remedies, and then, having recourse to tepid ones, the patient has been cured immediately. The liquor plumbi subacet. dilut. is now used at the Middlesex Hospital, tepid, and with general success. The strength of this application is to be gradually increased until it is doubled. It is in this complaint as in ophthalmia, that you cannot tell *à priori* whether cold or tepid applications will be the most successful; if therefore one fails, the other should be tried." (P. 35.)

Upon the subject of *Polypus Uteri*, Dr. Gooch has entered much more at length in his work "On some of the most important Diseases of Females," than he could be expected to do in a course of lectures: the following extract, however, conveys a good lesson to the young practitioner.

"The neck or stalk of the polypus is sometimes so small that, by slightly pulling it, the tumor comes away, and no hemorrhage ensues. When a ligature is applied to the neck of a polypus, which is itself insensible, the only pain occasioned by it is a slight sense of dragging; but if a portion of the uterus be included, the most violent pain will be produced. Dr. Denman once applied a ligature to a polypus uteri: he tightened it, and immediately the patient complained of pain; he relaxed the ligature, and again pulled it, and with the same effect. Being a cautious man, he loosened the ligature, and left it in the uterus; she died in a day or two. On examining the body, he found the polypus had grown from the fundus uteri, and by its weight had partly inverted the uterus, around which he had placed the ligature. Dr. Hunter in a case of this kind applied a ligature, and drew it tight; violent pain was produced; he slackened it, the pain ceased; he pulled it again, and agonizing pain was occasioned; he told the patient she must bear it; he gave her an opiate, and thus left her. The next day he found her with a thready and almost imperceptible pulse, bathed in cold sweats, with a cadaverous Hippocratic countenance. She soon died; and he discovered, on examination, that he had included a portion of the uterus in the ligature.

"Never tighten the ligature when it produces the pain which it would occasion on a part possessing sensibility; rather let the ligature be removed, and applied lower down, where the tightening of it is not productive of pain. It is not necessary to tie the polypus at the top of its neck; it is quite sufficient to tie it where it projects from the uterus into the vagina, supposing such to be the situation of it. The stalk will not grow again, but will gradually diminish and leave no vestige behind. All these cases are unaccompanied with diseased structure of the uterus. But there are some cases of fungus having a broad base and apparently of a malignant nature, in which the uterus itself is diseased. In such cases the ligature is of no use, as the fungus, if removed, would grow again, and the fate of the patient would be accelerated by its employment. When the uterus itself is diseased, in addition to discharges from this organ, and disorder of the general health, there is most commonly more or less pain, perhaps of a lancinating kind, aud the region of the uterus is also tender on pressure. In such cases the treatment must be directed chiefly to the preservation or improvement of the general health, together with the use of such palliatives as the symptoms may require. Malignant fungi of the uterus are rare; those of a benign character are the most frequent." (P. 54.)

In the lecture on the *Theory of Conception*, the following anecdote is detailed as a proof that it is not necessary that the woman should be sensible at the time of impregnation, or that she should have pleasurable sensations. "A maid at an inn, who was always thought to be virtuous, and bore a good character, began to enlarge in a way which excited suspicions of pregnancy; she solemnly declared that she never had connexion with any man: at length she was delivered, and was afterwards brought before a magistrate to swear to the father; but she repeated her former declaration. Not long afterwards a postboy related the following circumstance, that one night he came late to his inn, put his horses in the stable, and went into the house, and found all gone to bed except the girl, who was lying asleep on the hearthrug, and, without waking her, he contrived to gratify his desires. This shews that impregnation may take place without the knowledge of the female, or any excitation of sexual passion." We really think that no such inference ought to be drawn from so ridiculous a story, and we can hardly believe that Dr. Gooch related it seriously to his class.

In treating on the *Signs of Pregnancy*, it is observed, that "great stress is laid on the colour of the areola round the nipples, and in a first pregnancy (after which the brown colour, in a greater or less degree, becomes permanent,) it is a very important sign;" but in chronic uterine inflammation, Dr. Gooch has known this dark-brown colour produced, together with fulness and pricking pains in the breasts. The advice that is given to the practitioner as to the course he is to pursue, when consulted by unmarried women, who he has reason to suspect may be pregnant, is very sound, although rather quaintly put. "Do not believe one word they say; listen to them as you would to a jockey praising his horse." "Never rely upon the evidence of their tongues, but on that of their bellies."

*Œdema of the Labia Pudendi.* It occasionally happens that, during the latter months of pregnancy, œdema of the limbs and labia pudendi occurs. The labia may be enlarged to nearly the size of the head of a child; and as a proof that this circumstance may occasion blunders, we give the following candid statement of Dr. Gooch.

"When labour comes on, the external parts being in this state, the accoucheur, mistaking this for the child's head, may suppose so much of the labour to be over. When first in practice I made this mistake. I was called to a labour; the pains were severe: on examination I found a large tumor between the patient's thighs; and supposing it to be the head of the child, I requested the nurse to get every thing ready. Pain succeeded pain, but there was no advance of the shoulders. I then began to examine more minutely, and found the tumor to consist of an immense enlargement of one of the labia. Had I been more attentive at first, I should have discovered, as I did afterwards, that the skin on the outer side of the tumor was continuous with that of the upper part of the thigh, while the opposite side of the tumor only conducted the finger into the vagina. The labia may be thus enlarged without offering any considerable impediment to the delivery. In general, no treatment is required; but if, before labour comes on, the swelling occasions much pain, I would let out the effused serum by a puncture with a lancet. A lady had both labia greatly swollen, and very painful: she refused to have them punctured unless I made use of my eyes, and the tumor which presented itself absolutely astonished me. In half an hour after an opening had been made into the labia, the bed was drenched with serum, and the patient was perfectly easy. I have heard of these punctures exciting inflammation and producing gangrenous ulcers difficult to heal, as scarifications in anasarcous legs sometimes do; but I have never met with an instance of the kind myself, and am rather sceptical with respect to its having occurred." (P. 115.)

The lectures on *Uterine Hemorrhage,* and on the *Employment of the Forceps,* are particularly good, and merit the earnest attention of the obstetric student.

*Presentation of the Funis.* In this case there are three modes of treatment proposed: first, to turn the child, and deliver; secondly, to return the funis, and keep it up till the head descends, which will then effect the latter purpose; thirdly, to apply the forceps.

"If we detect a presentation of the funis, when the os uteri is nearly dilated, the membranes entire, and the parts in a relaxed state, no one would here hesitate to turn and deliver, as it may be done with ease and safety; and it will be best, in this operation, to employ the left hand, as the feet will, most probably, be in the posterior part of the uterus. If you attempt to turn under less favorable circumstances, nothing will be gained; for the delivery will be protracted, the life of the child consequently lost, and that of the mother placed in some danger. But suppose the os uteri dilated, the membranes ruptured, and the head, with the funis before it, descending into the pelvis; if possible, endeavour to return the funis, and keep it up until the head descends beyond it by a continuance of the pains. You may push up the funis by the side of the head with your fingers; but it requires to be kept there during several pains. I once had a funis presentation, when the life of the child was of great importance: I took a sponge, and pushed it up after the cord into the uterus; the sponge being of large size, kept up the cord, and the head descended properly. You may carry up the cord, and hang it on a limb, if practicable. On the continent they place the descended loop of the cord in a bag, in which it is pushed up beyond the head; and, when once up, the bag is too large to permit its descent. If the membranes are ruptured, and the head is descending rapidly, nothing can be done; but if the head is low down, and descending slowly, apply the forceps immediately, if other circumstances are favorable. If the funis has been down so long as to have become flaccid and destitute of pulsation, the child is dead, and the labour is to be left to nature." (P. 239.)

In *Puerperal Convulsions*, very free abstraction of blood is recommended. "Give me the lancet," says Dr. G., "and deprive me of all other remedies, and I will do more good with it singly than with all others, deprived of this, put together." Upon this subject, the advice and doctrines of Dewees appear to us more judicious than those of Dr. Gooch. The latter (presuming the lecture before us to be a faithful record of his opinions,) would have bled, and largely too, in every case, and without reference to the constitution of the patient, from a firm conviction that by this means alone could the formidable attack be relieved. "Bleeding is our sheetanchor, in whatever class of patients the disease may occur," says Dr. Gooch: Dr. Dewees, on the contrary, is persuaded, from what he has seen of this formidable complaint, that there is no one cause constantly operating to produce puerperal convulsions, and that there is not any one mode of cure applicable to all.*

The lecture on *Puerperal Fever* is good as far as it goes, but

* Dewees' Midwifery, p. 497.

the inflammatory species of the disease is alone described. Mr. SKINNER should certainly have brought up the lectures to the published work* of Dr. Gooch, in which is contained so much information of great practical import upon the *low type* of this disease.

We must not close our notice of this work without advising the obstetric student to give it an attentive perusal. We are aware that much will be expected from the lectures of Dr. Gooch; but it must not be forgotten that *viva voce* lectures, when printed, almost always disappoint: we criticise more severely with the eye than with the ear: the mode in which the lecturer enforced his points, the dexterity with which he kept the minds of his pupils to the subject before them, and his "face, cannot be printed." It would be unreasonable, then, to expect that these printed lectures should be as attractive as they were when delivered with the spirit and peculiar humour of the much-lamented Dr. Gooch; but as they contain the "pith and marrow" of his opinions and doctrines, they must be interesting to all students and practitioners in midwifery.

---

*A Practical Medico-Historical Account of the Western Coast of Africa: embracing a Topographical Description of its Shores, Rivers, and Settlements, with their Seasons and comparative Healthiness; together with the Causes, Symptoms, and Treatment of the Fevers of Western Africa; and a similar Account respecting the other Diseases which prevail there.* By JAMES BOYLE, M.C.S.L., Colonial Surgeon to Sierra Leone, &c.—8vo. pp. 423. Highley, London, 1831.

THIS is the best "practical medico-historical account" of the Western Coast of Africa that we have lately seen: and now, having furnished the publisher with a convenient extract for his advertisements, we must say that it is a volume of which we had rather speak in the superlative than in the positive degree; for we consider ourselves nearer the truth in declaring it the best, than if we should venture to pronounce it simply *good*. This, we confess, seems rather an ambiguous compliment, and, like the oracular revelations of the olden time, may be read to suit the wishes of the votary; and low, indeed, must be his developement of self-esteem who will not construe a doubtful passage in the most favorable light.

"Κροῖσος 'Αλυν διαβας μεταλην αρχην καταλυσει."

Memoirs on the subjects which swell the title of this volume are not numerous; and hence any account of a country and a climate so fatal to European health, and yet so important to European policy, cannot be wholly devoid of interest; and yet our author has contrived, with consummate skill, to render the details of both

* Account of the most important Diseases peculiar to Women; by ROBERT GOOCH, M.D. &c. 1829.

prolix and obscure. In wading through several hundred pages, we often stopped to ask ourselves what they meant? It was a question which frequently we could not solve, and we very much doubt if the writer could answer it himself. Had not the last page contained a list of "works by the same author," we should have supposed this written by an unpractised pen, and might have overlooked much of that looseness, and many of those redundancies that an older scribe should have pruned with an unsparing (which would have been a friendly) hand.

We would fain have given an epitome of between two and three hundred pages devoted to the subject of Fever, *local and climatorial*; but this would have been composing, not abridging an essay, for which our author has only provided the raw materials, and those not in the best state nor of the best kind: we shall, therefore, rest content with extracting those parts which will give the most information to our readers.

Mr. Boyle distinguishes between two African fevers (the local and the climatorial), not by their symptoms, but by their causes; and their successful treatment is made to depend upon a knowledge of causes which must be frequently unknown; for, however satisfactory *sol-lunar influence* may seem to our author, "verbum non amplius," it appears to us —. But our obtuseness shall not prevent him having the opportunity of convincing the public, nor will we disdain the office of conveying information to others, even if we ourselves remain uninformed.

"Agreeably to the arrangement proposed in the introduction, respecting what is conceived to be a very necessary and even a vital distinction, that is, the difference between similarity of symptoms and actual consequences, the question entitled to primary consideration is, an inquiry into the causes of climatorial bilious remittent fever, as it ordinarily occurs to the crews of ships of war, or to those of other ships, which have not been at all, or but very little, exposed to local influence.

"In this inquiry much candour is called for, and it is right at the outset to explain, that it is not by the symptoms alone that the novitiate practitioner upon the coast, or even the experienced local practitioner there, is to judge of the distinctions in this fever, which are held to be so important. It is circumstances, and circumstances alone, that should determine as to the original causes, and upon which, as it will hereafter be endeavoured to be shewn, the treatment should be made most materially to depend. This, at first sight, may appear to be a somewhat unnecessarily minute view, but it proceeds from a positive conviction, founded upon experience, that it is both proper and advantageous to institute the closest distinction possible with respect to the causes of this fever. Before proceeding further, however, it is incumbent to express the fact, that, when the patient has been exposed to the influence of the land, the causes of his fever may be of so mixed a character, between climatorial and local excitants combined, as to put it beyond the power of the practitioner to draw a distinct line of demarcation. This observation, of course, refers only to a difficulty of occasional occurrence, and one which forms the exception to a rule that appears to be at once just and general, and therefore worthy of serious attention. This difficulty, or mixture of causes, having thus been noticed, a particular inquiry into it may, with propriety, be deferred until the nature of the simple climatorial fever has been investigated: that investigation may be at once entered upon.

"Great caution is necessary to enable the practitioner to distinguish between the climatorial bilious remittent fever and the simplest form of the African local bilious remittent fever, which might be otherwise easily confounded; for, as the cruizing ships of war upon the coast, though not to the same extent as merchant ships trading there, are exposed to more or less local influence at uncertain periods, varying from circumstances, after their arrival upon the station, it may sometimes be very difficult to say whether the fever occurring depend on climatorial or local causes, or upon both combined." (P. 73.)

"Sol-lunar influence is powerful in the production of fever on the western coast of Africa, and indeed in all parts between the tropics. Many instances have been known of men, whilst at work under the rays of the sun, dropping down as if shot; and that without any previous threatening symptom or habit of indiscretion; and also men who, to avoid the closeness sometimes experienced in sleeping between decks, have slept on the upper deck, without the knowledge of the officer on watch, thus exposing themselves to the apparently harmless beams of a brilliant moon, have often been known to be suddenly affected with fever. The rapidity of the latter attacks precludes the thought that they were attributable to damps or dews that might be falling in the night, and which, indeed, are also common causes of fever, but not so immediate in their consequences." (P. 75.)

"It is unnecessary, however, to remark further respecting the complex character of this fever, which is here to be considered in its more simple form, and not as a contagious disease; a character it only assumes under particular circumstances." (P. 77.)

The climatorial bilious remittent fever is described as being ordinarily characterised by severe headach, pain at the pit of the stomach, retching or vomiting, with a costive state of the bowels; sometimes vomiting of green bile, great heat of skin, suffused eyes, and thirst, the tongue being generally more or less furred; usually, also, pains in all the joints, and tottering of the limbs; the pulse varies from ninety strokes in a minute to 120 or 140. On minute inquiry, it will generally be found that the patient is suffering from severe pain in the back or loins; and if the face do not happen to be flushed at the time, it will have a livid hue, its features having a downcast appearance, and there being, in all probability, a dark areola around the eyes. Under these latter circumstances, the pulse will be less developed, nor will the skin attain to so high a temperature. Most attacks will be preceded by a sense of chilliness, and attended with loss of appetite; and in all the greater part of the symptoms described will soon set in, and establish the true character of the disorder." (P. 77.)

The details of several cases then follow, in which the patients were largely bled: they are given to prove that causes, not symptoms, are to distinguish these forms of fever. The author remarks,

"The four foregoing cases have been selected from the journal of cases occurring on board his Majesty's ship Cyrene, in 1823, for the purpose of affording a contrast between such cases as may be termed climatorial endemic, and such as, in contradistinction, have been designated local endemic. The first and last are what may be denominated local bilious remittent fever; and the second and third are simple climatorial fever, as may be seen upon a reference to the causes. Though not perceptible to the eye at the commencement of the cases, the difference in the real nature of them existed at their outset, and the treatment in all of them, as far as regards bloodletting, being the same, the result, consequently, unhappily varied in proportion to that difference.

"Whilst in the harbour of Sierra Leone, the Cyrene had fifteen cases of climatorial and local fever on board, all of which were treated by bloodletting, and the cases No. 1 and No. 4, recorded as having had local in addition to climatorial causes to contend with, were the only two that proved fatal.

"Such is the fact with respect to general bleeding, that, if the author, with the additional experience he has acquired since he was surgeon of the Cyrene, was called upon now to treat patients so circumstanced as were Mr. Hunter and Jones, he would not think of bleeding them from the arm. Hereafter strong practical grounds will be advanced in justification of this difference of practice in cases of apparently the same disease, occurring on board the same ship, and at or about the same time; but with the all-important difference of their having been excited under different circumstances." (P. 85.)

Of the second form of African fever the author writes in a similar strain; so that, forgetting the "non omnibus," we are tempted to exclaim "Facies una, nec diversa."

"As the endemic, or local bilious remittent fever, rarely fails to attack the British settler on the western coast of Africa, within the region bounded by the equator and the northern tropic, the consideration of its causes, its nature, and its treatment is a matter of great importance, and may with propriety immediately succeed the inquiry into the climatorial fever.

"The endemic is often wonderfully like the climatorial fever in its appearance; and, indeed, so much so, that a young practitioner might be induced to conclude that the adoption of the different names was only the creation of a distinction without the actual existence of a difference. Such an error, however, as that, experience in treatment would soon detect, but rarely with sufficient alacrity to prevent the occurrence of fatal results.

"The great distinction between these two forms of disease is simply this, that, although the same symptoms may appear in each form, yet the same treatment will not apply, but must depend upon the peculiar or specific nature of the cause of the fever, which should be most scrupulously inquired into, and viewed as the only safe guide by which the treatment is to be regulated.

"*National Predisposition to the African Endemic, or Local Bilious Remittent Fever.* The exclusive predisposition of the natives of the British isles, and those of other cold countries, to the African endemic, or local bilious remittent fever, which is certainly as important as the facts connected with it are curious, has never hitherto been noticed by any previous practitioner, either in the colony of Sierra Leone or on the western coast of Africa: at least, if the subject has attracted attention or remark, not even the most distant hint respecting it is to be found in any of the extensive manuscript writings which have been examined of the medical officers in the public service, who have been employed on the western coast of Africa."

"An almost daily experience during a period of four years in Freetown, afforded ample means for observation respecting the point under consideration, and, as is generally the case, the observance of particular facts naturally directed the mind to a consideration of the causes whence these originated. A conviction soon established itself on the mind, resulting from this inquiry, that all persons from cold countries are predisposed, or liable, one day or other, to the African local bilious remittent fever; whilst southern subjects, or those of a warm latitude, are, almost to a man, exempt. Among the English, Irish, Scotch, North Americans, Dutch, and Swedes, there seems but little, if any, difference in the existence of predisposition; but it may be stated, with confidence, that the ratio of mortality is greatest, in proportion to numbers, amongst the people of the two latter countries than it is amongst those of the four preceding ones. It seems, indeed, and as if in pursuance of the fact that the subjects of cold countries are almost exclusively prone to the local bilious remittent fever of Sierra Leone, that consequently, and upon the same principle, the colder the country from which the subject comes, the more dangerous his case. (P. 120.)

Although at first much prejudiced in favor of (profuse?) bleeding, subsequent experience made him give up this practice; and he agrees with other practitioners, that, although the African fever "in its character much resembles the bilious remittent of the East and West Indies," still, "on the coast of Africa, patients do not bear the loss of that quantity of blood which, by proper after-management, acts almost as a specific in the former countries." He says,

"Among a great many fever cases under my care, were seven of the crew of the Thomas Gelston, a merchant ship, which had been about eight weeks on the coast, and loading with timber up the Sierra Leone river. The cases were brought on shore, and lodged in the same house. The captain, having requested my attendance, accompanied me, and in his presence I blooded six, in whom the febrile symptoms were most marked. In the end, the whole of the seven died; but the most delicate, and the one not blooded, outlived the strongest of the others for the space of about twenty-four hours. Apprehensive that this fatality might have depended upon some uncommon specific cause, I continued the system of bleeding still longer, limiting its application to such cases as had only very recently been attacked, but with very little success. At length, being conscientiously forced to abandon a practice to which I had, in a measure, been wedded, as well from my own previous experience in the treatment of other tropical fevers as from the concurrent testimony of the profession generally, I was induced to seek for an explanation by a reference to localities. I had now the advantage of seeing and treating fever, as well on board of ship as on shore; and I had observed that a similarity of symptoms, under these different circumstances, did not justify a similarity of treatment. I was also further convinced that the cases which occurred amongst seamen, who had but little or no communication with the shore, were more sudden in their approach, that the symptoms were more intense in their appearance, and that they evinced a more inflammatory character than did those of Europeans even but a few days located on shore, or such subjects as were employed in hard labour during the same period up the river. I thus came to the conclusion, in short, that, although occasionally difficult to distinguish between them, two forms of bilious remittent fever are to be recognised on the coast of Africa; and these, it may be seen, I have distinguished by the epithets climatorial and local." (P. 132.)

Of the "diseases of frequent occurrence upon the western coast of Africa, but which are not peculiar to that coast," viz. pneumonia, catarrh, gastritis, enteritis, splenitis, colic, diarrhœa, cholera morbus, ophthalmia, variola, vaccina, apoplexy, paralysis, mania, melancholia, dyspepsia, syphilis, gonorrhœa, phagedænic ulcer, and tetanus, although the catalogue is fearful, it might easily have been enlarged by further extracts from a nosology, without tempting us to spend much time on the review; for we do not find one fact of importance recorded concerning those above named; and, indeed, here, as in many other parts of his work, we wish the writer had distinguished between *truths* and *truisms:* and as to the "diseases more especially tropical, the cases of "Craw-craw," (scabies,) seem, by the account, to be so like psora, that we really itch to call them so. The treatment of Frambæsia by caustic, as recommended by Mr. Bell, must form our concluding extract:

"'Frambœsia, or yaws, is an affliction from which the inhabitants along the seacoast suffer: it is said by themselves to be hereditary, as I observed the spleen to have been. At present (says Mr. Bell,) I have a case under cure, whose face is covered with excrescences resembling raspberries: he had a phagedænic ulcer on the preputium; the perineum was studded with similar excrescences to those upon the face, and there were several over the legs and thighs. The symptoms he laboured under when I first saw him, indicated great derangement of the digestive organs, to which I directed my treatment. I commenced by giving small doses of rhubarb every day, and a five-grain bluepill every second night. His stomach and bowels have now acquired tone; the tongue, which in the first instance was foul, is now clean, and the youth is getting lusty.'

"In his capacity of surgeon to the mixed Commission Courts, and likewise to the Liberated African department, the writer of this volume had extensive opportunities of seeing and treating this disease. Amongst the many thousands of slaves that of late years arrived at Sierra Leone, it frequently occurred, and he can speak confidently of the success of a very simple treatment, which was aided by a favorable change of diet and cleanliness on the part of the patients.

"The treatment of the people in question consisted simply in their being regularly and carefully washed every morning, after landing; on their being each supplied with a clean new dress; their having alterative medicine occasionally administered to them; and in the affected parts being touched daily with caustic.

"To this last remedy alone the slavedealers generally trust in the treatment of the complaint, and to them we are indebted for this piece of knowledge. Undoubtedly, this local application, and the adjuvants just named, may be depended upon as measures speedy, safe, and certain in their curative operation over this disgusting disorder." (P. 390.)

---

(*Published in Monthly Parts.*) *The Cyclopædia of Practical Medicine.* Edited by John Forbes, m.d., Physician to the Chichester Infirmary, &c.; Alexander Tweedie, m.d., Physician to the London Fever Hospital, &c.; John Conolly, m.d., late Professor of Medicine in the London University. *Part I.*—Royal 8vo. pp. 112. London: Sherwood; Baldwin and Cradock; and Whittaker.

It has long been a subject of surprise to us that a work upon the plan of that before us had not been undertaken in this country. The success with which the French and Germans have published their Medical Dictionaries, of various sizes and various degrees of merit, would, we should have thought, have emboldened *our* booksellers to seize upon any offer of the kind that was made to them, with a more than ordinary degree of confidence. We have reason to know that editors have not been wanting, but that some of the leading houses in the "Row" have hitherto been shy of the undertaking. Our own opinion has always deen decidedly in favor of such a publication, provided, indeed, it came out under the authority of men who were known to the profession to possess adequate ability for their task. To the "Editors" of this Cyclopædia no exception can be taken; the list of "Contributors" also contains many good and honoured names: there is none we would willingly strike out, but there are certainly some we should have added, if to us had fallen the selection.

Of the foreign works of this kind, the one with which we are best acquainted is the "Dict. des Sciences Medicales," in sixty volumes: here there is much to imitate, and much to avoid. One great fault of this Dictionary we hope the conductors of the "Cyclopædia" will carefully avoid. Many of the articles are rather erudite and laboured statements of old and obsolete opinions, than descriptions of the doctrines and practice of the present day. We would not have it imagined that we would neglect the labours of our predecessors, but we would expend as little time and space as possible in the relation of what are now known to be their errors. In the Dictionnaire des Sciences Medicales, as much pains appear to have been taken to go through a dry detail of detected blunders as to expound the best doctrines of the day: in short, the writers of many of the articles appear as anxious to tell us what is now known to be false, as what is now known to be true; and this, in them, lamentable fault arose, we conceive, from an over-anxiety to display their intimate acquaintance with all the old "medical classics," whether good or bad. As yet we have no reason to fear that the "Cyclopædia" will be loaded with this learned rubbish: the articles in the first part of it, at least, are free from any such redundant matter. The fancies of olden times are very properly passed by, that more honour may be done to established facts and doctrines.

Of such a work our readers will not expect a formal analysis: we shall briefly mention the different articles contained in the first part, and extract, as specimens, occasional paragraphs from them.

The first article, "*Abdomen* (*Exploration of the*)," is from one of the editors, Dr. Forbes: it contains a satisfactory account of the different methods employed by physicians for *exploring*, or physically examining, the external parts of the abdomen, with the view of discovering or discriminating the diseases of the viscera contained in this cavity. The different *regions* of the thorax and abdomen are illustrated by plans designed and executed by Mr. Paxton, of Oxford.

The second article, "*Abortion*," by Dr. R. Lee, is defective, on account of its brevity: it is much more than a definition of the word, and infinitely less than a satisfactory account of so important a subject. The learned author states, that "by far the most frequent cause of abortion is in the product of conception itself, viz. in a diseased condition of the fœtus, or its involucra, by which it is deprived of life, and afterwards expelled from the uterus like a foreign body. The blighted ovum is thrown off from the parent, as fruit which has become withered is separated from the branch of the tree on which it has been produced." We do not remember to have seen this opinion so strongly insisted upon before, and *we* are still inclined to believe that abortion is much more frequently independent of, than dependent upon, disease of any part of the ovum itself, although we willingly admit it as an occasional cause. A well-known German writer, Jörg,* goes further than any other authority we know in support of Dr. Lee's opinion, and adduces many facts and arguments to shew that abortion may depend upon the causes he assigns. Dr. Lee says,

"We have examined numerous ova which have been prematurely expelled, and in many of these, where no disease was obvious at first, some morbid state of the membranes, placenta, or embryo itself, has been detected, sufficient to account for the accident, wholly independent of any constitutional or local affection of the mother. Sometimes the chorion has been thickened, opaque, and extremely irregular, or lobulated, on its internal surface. The amnion in some cases has undergone similar changes, so that the healthy appearance of the involucra has been entirely lost. A collection of serum, or blood, has not unfrequently, also, taken place, between the chorion and amnion. The placenta, in some cases of abortion after the third month, has been hard, like cartilage, small and imperfectly formed, with calcareous particles deposited in its substance: in others, the placenta has been unusually large, and its vascular structure has been changed into a soft yellow fatty substance; or hydatids have been developed in its tissue. The umbilical cord, in these instances, has been remarkably slender, and the fœtus has appeared to perish for want of a proper supply of nourishment; and not from any defect in the organization of its internal parts.

"The brain of the fœtus, or the thoracic or abdominal viscera, may all undergo various alterations of structure incompatible with life; and where the life of the fœtus is extinct, it becomes an extraneous body; expulsive efforts on the part of the uterus are usually soon set up, and abortion ensues as the necessary consequence. When the ovum is healthy, it adheres to the uterus with great force; but when diseased, the slightest shock to the mother, the most trifling mental affection, is sufficient to cause it to be expelled. Women have had the bones of the extremities fractured during pregnancy, and have suffered other grievous injuries, without miscarrying. A woman mentioned by Mauriceau escaped by a window from the third floor of her house when on fire, and in her fall to the ground fractured her arm, yet abortion did not follow. The case of a young woman with a narrow pelvis is related by

* Handbuch zum Erkennen und Heilen der Kinderkrankheiten, Zwölftes Kapitel.

Madame Lachapelle, who threw herself into a deep pit, and suffered injuries of which she subsequently died, yet the fœtus was not expelled." (P. 11.)

Dr. Tweedie furnishes the next article, "*Abscess.*"

Dr. Marshall Hall treats on "*Abstinence:*" and here again we must complain (and such a complaint is rather complimentary than otherwise,) not of what is said, but that too little is said upon a subject which is of the very utmost importance, as has been recently shewn by M. Piorry, whose essay on it we gave in our first volume for 1831. The following cases, quoted by Dr. Hall, are instructive.

"Interesting cases of abstinence are recorded with the full detail of symptoms, by Dr. Currie (*Medical Reports*, Ed. 4, vol. i. p. 304,) and by Dr. Willan (*Miscellaneous Works*, by Dr. Ashby Smith, p. 437.) To these cases we refer our readers; extracting only what will be sufficient to portray the usual character of the late symptoms in the case of inanition, and lead to the due distinction between the symptoms of disease of the nervous system which arise from it, and those which depend upon a primary disease of the brain itself; for the case of extreme abstinence or famine affords a remarkable instance of the singular effects of exhaustion, and their similarity to those of diseases of a totally opposite nature and origin.

"Dr. Currie describes the effects of inanition in a case of obstructed œsophagus, as involving the following circumstances:

"The patient's age was sixty-eight: the obstruction in swallowing gradually increased from the beginning of August, and on the 1st of November it was complete. The following plan was adopted: each morning, at eight o'clock, he had a clyster consisting of eight ounces of broth, two yolks of egg, and forty drops of laudanum; this was repeated at three P.M. and at nine P.M. with thrice the quantity of laudanum. Previously to the evening draught, a bath was used, consisting of one fourth part of milk, and three fourths of water, at 96°. In a few days the clysters were augmented in quantity, and eight ounces of wine were added, with an increased dose of laudanum. This plan was continued until the 2d of December. The rectum then ceased to retain the clysters, and their employment was relinquished. In spite of these plans the emaciation was rapid: the patient had weighed 240lbs. when in health; on the 20th of November he weighed 154lbs., and on the 25th, 149lbs. only.

"The pulse, during the month of November was that of health; on the 1st of December it became small and frequent; on the 2d, it was still more frequent, though stronger, with delirium, and the case resembled the last stage of fever. 'During this delirium, a perpetual and indistinct muttering occurred, with great restlessness and agitation; the surface and extremities were sometimes of a burning heat, sometimes clammy and cold. The eyes lost their common direction, the axis of each being turned towards the nose. In this state, however, the sensibility of the retina was not impaired, but rather increased, for he screamed out on the light of the window being admitted, to which before he had been accustomed. At this time also the sense of touch seemed more than usually acute, for he appeared disturbed with every accidental breath of air. The delirium and derangement of vision commenced nearly together, but we observed the derangement of vision first. On the 1st of December, he complained that he sometimes saw double; but it was not till the succeeding day that any considerable incoherence of mind was observed. The pulse became feeble and irregular on the 4th; the respiration, which had been singularly undisturbed, became laborious; the extremities grew cold; and in ninety-six hours, after all means of nutrition as well as all medicine had been abandoned, he ceased to breathe." During the first periods of this case there was little complaint of hunger or thirst; there was a stool, of a solid form, pale colour, but natural fœtor, with each fifth clyster. The spirits were even, and the nights good.

"Dr. Willan's case was one of monomania. He visited the patient on the 23d of March, 1786, on the sixty-first day of his fast. His emaciation was extreme, and he laboured under great imbecility of mind; the eyes were not, however, deficient in lustre, and the voice remained clear and sound. He was directed to drink a pint of barley water, and two cups of panada, which agreed. He had a little fever in the first part of the night, but slept better than usual. On the 24th he had some mutton-tea; the pulse was small and temperate. On the 25th he took a pint of milk for breakfast, a pint of mutton-broth boiled with barley for dinner, and as much rice-milk for supper, at his own request. He had considerable cravings for food all the day. In the morning of the 26th he drank tea, and ate a great quantity of bread and butter, which he got from off the table in the nurse's absence. Some time after he became sick, and vomited once or twice without much straining. About noon he had a figured natural stool, and presently after two or three loose motions. His skin was always dry. In the evening he was apparently much better; the pulse ninety and firmer. On the 27th he took a little light bread pudding at dinner, and had two eggs for supper; he rested well, and was cheerful. On the 28th he seemed better; he had not slept well, however, nor had a stool. On the 29th the scene was entirely changed: he began to lose his recollection in the preceding evening; and before midnight became quite frantic and unmanageable. His pulse was increased in frequency, with considerable heat on the skin, and tremors. He continued raving, and talking very incoherently, as he had done during the night. A strong purgative draught, and two clysters administered in the course of the day, produced but little evacuation.

"He remained nearly in the same state of mind as above mentioned, scarcely ever sleeping, and taking very little nourishment, till the 2d of April, when a considerable quantity of loose feculent matter was brought away by a clyster. Soon after he became sullen, and took no notice of what passed about him. He was removed at this time into the country, so that he was not seen again till the 6th of April. He appeared then emaciated to a greater degree, if possible, than at the first. His pulse was small and feeble, beating 120 strokes in a minute. April 7th and 8th he took whatever nourishment was offered him; knew those around him, and spoke sensibly, but faintly. On the 9th, in the morning, he died, quite exhausted.

"The case of abstinence affords a beautiful illustration of the fact, that cerebral symptoms, resembling those of increased action, do also arise from the opposite state of the brain and of the system. The effects of exhaustion frequently simulate phrenitis in adult age, and hydrocephalus in infancy, and it requires the utmost attention and sagacity to distinguish them." (P. 22.)

The article "*Acne*" might, we think, have been more briefly dismissed, and thus greater space could have been spared for other and more important subjects which have been too scantily treated.

Dr. Roget contributes several interesting and well-written pages on "*Age*," although we do not fix upon any particular portions to transfer to our Journal.

Under the head of "*Air* (*Change of*)," Dr. James Clark offers many good practical remarks on the principal circumstances which should guide the practitioner in prescribing change of air, in order that the patient may derive all the benefit from the remedy of which his case admits.

The next articles are "*Alopecia*," by Dr. Todd; "*Alteratives*," by Dr. Conolly; and "*Amaurosis*," by Dr. Jacob."

Dr. Locock is too brief on "*Amenorrhœa*," but his remarks are practical and correct.

Dr. Marshall Hall's article on "*Anæmia*," we give without abbreviation, as it appears to us particularly interesting.

"Anæmia. This term is derived from α, privative, and αἷμα, blood, and means, therefore, a deficient quantity of the circulating fluid, or bloodlessness.

"The hæmatosis, or formation of the blood, is begun in the stomach and completed in the lungs. A morbid state of any one of the organs or functions which concur to effect the hæmatosis may lead to a state of anæmia. It is in this manner that we have a defective state or quantity of the blood in some forms of dyspepsia, especially chlorosis. It is in this manner, too, that the workers in certain coal-mines have, from a deficiency in the pulmonary function, been struck with anæmia. A similar state of anæmia has also arisen from disease in some particular organ forming a part of the class of those which contribute to hæmatosis; a case, the exact seat of which is often obscure. It is scarcely necessary to add, that a state of anæmia is also the immediate result of losses of blood, either artificially or by natural hemorrhage: these are precisely the cases to which the term anæmia has been variously and too indiscriminately applied. M. Andral describes anæmia at great length, and has distinguished different forms of local anæmia, or anæmia of the individual organs.

"The case of chlorosis, to which the term anæmia has been applied adjectively, will be treated of distinctly in this work. So will the case of anæmia from actual loss of blood. In the present article we purpose to describe, 1, that form of anæmia which has occurred in coal-mines; and, 2, that other form of the disease which has an obscure origin in the morbid condition of some organ contributing to the hæmatosis: 3, to these will be added the view taken of anæmia by M. Andral, and especially the case of local or topical anæmia.

"General anæmia is denoted by extreme paleness, especially of those parts which, being covered by the thinnest and most transparent investing membrane, expose the condition of the blood underneath them, as the prolabia, the tongue, the gums, the internal surface of the cheeks, &c. The face, the hands, and the general surface, are pallid, and slightly waxen or icterode in their hue. There are vertigo, faintishness, palpitation, and an impaired action of the organs generally, especially of the stomach and bowels, digestion being deranged, with flatulency, constipation, &c.

"1. The following description of the anæmia of the coal-miners of Anzain is translated from an account given by M. Chomel, in the article Anémie, of the Dictionnaire de Médecine:

"All the workmen employed in one of the galleries of the coal-mines of Anzain fell sick in the summer of the year 11, although that gallery had been wrought for some time. The workmen of the adjoining galleries escaped, although the only observable difference was that they were less extensive, and the ventilation less difficult.

"The disease began with violent colic, with tympanitic distention, black and green alvine evacuations, to which were added dyspnœa, palpitations, and great debility. These symptoms subsided in the course of twelve days, and then those of anæmia appeared: the face assumed the colour of wax rendered yellowish by time; the blood-vessels had so entirely disappeared, that not a trace of them could be found where they are usually most obvious, as in the conjunctiva, eyelid, internal mouth; the arterial pulsations were feeble: these appearances continued even during attacks of fever, which came on accidentally in some of the patients: extreme feebleness; great anxiety; slight œdema of the face; palpitations; shortness of the breath on the slightest effort; perspirations: such were the symptoms: although the appetite was not lost, yet the digestion was imperfect, and there was a progressive loss of flesh.

"This state continued for six months or a year, sometimes terminating in death, preceded by the symptoms which first appeared.

"The Société de l'Ecole de Médecine was consulted. Four patients were conveyed to Paris, and placed under the care of M. Hallé. A nutritious diet, infusion of hops and gentian, the 'vin antiscorbutique,' were conjoined with mercurial frictions. During this treatment one patient died; on examination, the arteries and veins were found destitute of coloured blood, and containing only a little serous fluid; incisions through the muscles gave rise to no flow of blood, except a little from those of the thigh. The absence of blood, which was in accordance with the external phenomena, led to the disuse of mercury, and to the substitution of the internal use of iron, (limaille porphyrisée,) in the dose of a 'gros' daily, under the form of opiate, with tonics. In eight or ten days there was an evident amendment: several veins appeared under the skin of the fore-arm; the digestion improved; the shortness of breath diminished. On each successive day the patients pointed out, as discoveries, new veins, which had not been perceived the day before. The amendment was progressive, and quite complete when these men returned home.

"Similar morbid appearances were observed in some of the patients who died at Anzain; and the same mode of treatment proved successful both there and at Dunkerque, whither some of the sufferers had also been conveyed.

"The want of sun and the want of air seem to have been the causes of this singular malady. These important agents appear to be essential to hæmatosis. The effects of the want of blood are illustrated in an interesting manner. But the consideration of this point is reserved for a distinct article.

"2. The origin of the second form of anæmia is more obscure. We shall illustrate the subject by an abstract of a case published by Dr. Combe, in the Transactions of the Medico-Chirurgical Society of Edinburgh.

"Dr. Combe first visited his patient in July 1821. He looked like a man recovering from syncope: the symptoms were pallor, languor, breathing easily hurried, pulse eighty and feeble, tongue covered with a dry fur; the bowels relaxed, the stools dark and fetid; thirst, want of appetite, rejection of food; no pain; no detectible organic disease. The patient was forty-seven, married, of regular habits, and engaged first in agriculture, and then as a servant to a corn-merchant; he was never blooded. These symptoms had stolen upon him during two months; he said his head troubled him; and the feet were œdematous. Some tonic medicine, a mild nutritious diet, and wine were prescribed. Afterwards iron, mercury, opiates, and astringents were given. Towards the end of September he tried a sea-voyage, and a chalybeate spring. He died in January, with all the symptoms of hydrothorax. On examination, not a drop of blood flowed on dividing the scalp; the dura mater was moist, and displayed few vessels, and those empty; near the vertex, to the left, there was an ossification; the pia mater was pale, its blood-vessels containing a pale serum, and air; a slight effusion under the arachnoid. The brain was soft and pultaceous, with few vessels, and little difference of colour between the cineritious and medullary portions; the ventricles contained two drachms of serum; and about two ounces were found at the basis. The lateral sinuses were moderately filled with pale fluid blood; the arteries at the basis were empty. In the thorax there were three pounds of a lemon-coloured serum; the lungs were of a pale grey, without any mark of gravitated blood. The pericardium contained an ounce of serum. The heart was pale; the right ventricle contained a pale coagulum; the left was empty. The inner coat of the aorta was of a fine red colour. There was some moisture in the abdomen; the liver was of a light brown colour; the spleen alone was of its natural colour, and soft. The arteries were universally empty, as were the jugular and femoral veins; the lower cava alone contained any blood.

"The whole case, and, indeed, the whole subject, is exceedingly obscure. It does not appear that any examination of the thoracic duct was made, which is much to be regretted.

"3. We now proceed to notice the different forms of local anæmia; and, in doing so, we shall chiefly draw upon the recent work of M. Andral. (Précis d'Anatomie Pathologique, p. 73.)

"M. Andral traces the cases of local anæmia, 1, to a diminution of the principal artery; 2, to certain affections of the nervous system; for instance, may not the stomach become anæmious like the cheek? 3, to the hyperæmia of other organs; 4, to a previous state of hyperæmia in the organs affected with anæmia; 5, lastly, without being able to trace it to any of these causes, a state of anæmia is sometimes seen in some particular organ. M. Andral adds that he has observed this state of anæmia particularly in the brain, the heart, the liver, the stomach, and some other parts of the alimentary canal, and some of the voluntary muscles.

"With a state of anæmia of an organ, that of atrophy is sometimes conjoined.

"With anæmia of the membranes, we often observe an augmentation of their secretions; this is seen in the serous and mucous membranes, and in cutaneous textures.

"When anæmia is induced in an organ by the sudden obliteration of its principal artery, as by ligature, gangrene is the result.

"M. Andral concludes this subject by the important remark, that the symptoms of the anæmia of an internal organ are sometimes observed during life; convulsions and delirium arise from anæmia, as well as hyperæmia, of the brain. Similar states of the stomach may lead to their peculiar symptoms respectively.

"It is, indeed, quite plain that the state of anæmia, of which the actual loss of blood is one of the most frequent forms, leads to phenomena which require to be carefully distinguished and appropriately treated." (P. 71.)

"*Anasarca*" is well described by Dr. Darwall. In distinguishing inflammatory from asthenic dropsy, which is not always so easy as it might abstractedly appear, Dr. Blackall lays much stress upon the presence of serum in the urine; but upon this point Dr. Darwall makes no remark, having no dependence upon it as a guide of practice.

"That it is never present without inflammation is, perhaps, true; but to make it really a test, the converse ought also to be true, viz. those dropsies in which it does not contain serum ought never to be conjoined with inflammation. This, however, is by no means the case; and our experience accurately coincides with Dr. Crampton's. 'In many of those,' says this author, 'which appeared to me to require the prompt use of the lancet, the urine did not coagulate. Under this impression, I ceased to draw any practical inference from that appearance;' and he adds in a note, 'Dr. E. Percival, who was my predecessor as one of the physicians to the House of Industry, mentions that the result of his experience on this subject fully coincides with mine. After he had tried dropsical urine by the test of coagulation in a number of cases, he at length lost all confidence in the test, either as an invariable evidence of inflammation, or as a guide of practice. His statement is likewise confirmed by the additional testimony of Dr. Reid, who acted as a clinical clerk at the House of Industry at the time those experiments were made.' We would much rather, therefore, recommend that reliance be placed upon general symptoms than upon the state of the urine only, which, at the most, can only be regarded as an auxiliary to our diagnosis." (P. 77.)

To prove that acute anasarca is not always an inflammatory disease, the following extract is taken by Dr. D. from Dr. Bateman's Reports of the Diseases of London. "The patient was a middle-aged woman, in previous health: she was thrown into a state of extreme fright and alarm, on discovering in the evening that she had lost her little store of money, the savings of several years, and the next morning she was anasarcous from head to foot. By tonics, combined with diuretics, the disease was speedily removed."

On "*Angina Pectoris*," Dr. Forbes gives an excellent article. For the purpose of fully corroborating his opinion that the pain in the paroxysm of angina has its primary and principal seat in the heart or annexed great vessels, the following brief outline is given of the results obtained on dissection, in some of the principal cases recorded by authors since the time of Dr. Heberden.

"A. *Results relative to the existence of organic disease in general.*

| | |
|---|---|
| Total number of cases of which dissections are given | 45 |
| Of this number, there was no organic disease (except obesity) in | 4 |
| Organic disease of the liver only, in | 3 |
| Organic disease of the heart or great vessels, in | 39 |

"B. *Results relative to the nature of the organic affections of the heart and great vessels.*

| | |
|---|---|
| Total number of cases in which there was organic disease in the heart or vessels | 39 |
| Of this number, there was organic disease of the heart alone, in | 10 |
| Organic disease of the aorta alone in | 3 |

Organic disease of the coronary arteries alone, in 1
Ossification or cartilaginous degeneration of the coronary arteries in . . . . . . . . . . . . 16
Ossification or other disease of the valves in 16
Disease of the aorta (ossification, or dilatation, or both), in . . . . . . . . . . . . . 24
Preternatural softness of the heart* in . . 12

"If we consider it as proved, by the foregoing statements that the heart and its great vessels constitute the seat of the pain and all the principal phenomena of the paroxysm of angina, it still remains to be inquired what is the nature of this pain, and what are its immediate causes, or the circumstances on which it immediately depends. And there seems no great difficulty in coming to a satisfactory conclusion in this matter, if we keep in view, 1, the facts recorded relative to the frequency of structural disease in the heart and aorta in angina; 2, the characters of the pain itself, its instantaneous invasion and cessation, its violence, its short duration, and its complete disappearance for long periods of time; 3, the nature of the actions of the heart and great vessels in health; and, lastly, the nature and causes of painful affections in other parts of the body.

"We have no doubt that the pain, and other principal phenomena characterising the paroxysm of angina, may acknowledge different causes and depend on different conditions of the affected parts, in different individuals, and in the same individual at different times; just as we find in other parts of the body, more particularly in parts possessing a structure and functions somewhat analogous to those of the heart, namely, the hollow viscera possessing muscular tunics, and destined to convey fluids. The stomach, the intestines, the bladder, the gall-ducts, ureters, &c., are all subject to sudden paroxysms of severe pain, frequently originating in very different causes, yet very similar in their external character. In the stomach or bowels, for example, we may have gastralgia or enteralgia, of the same general appearance, from pure muscular spasm; from matter irritating the internal membrane; from simple neuralgia, the source of irritation being at the origin of the nerves; from over-distention; &c.; and each of these may exist with various organic diseases of the part, or without any organic disease; and two or more of them may coexist. It is reasonable to suppose that painful affections of this kind will much more easily be produced in a diseased organ than in a sound one; and indeed experience proves that, although almost all organs are liable to severe pains, from some unknown temporary condition of the nerves themselves, usually termed neuralgia, yet that they are much more liable to such painful affections when diseased in their structure, and consequently disordered in their action or enfeebled in their powers. From what has been already

* In a great many cases the consistence of the heart is not noticed.

stated of the frequency of structural disease of the heart and aorta necessarily disordering their action, it will readily be understood how easily pain may be induced in these parts under various circumstances, and from many different causes; and it seems equally intelligible how similar pains may originate in the same parts, under particular circumstances, even when no structural lesion exists in them. In this, as in so many other cases, authors have frequently failed in accounting for the phenomena, because they took too confined a view of the subject, and were anxious to establish a greater simplicity in the production of the symptoms than nature acknowledges.

"Of the intimate nature of the pain in the paroxysm of angina, we know nothing; but we know no more of the nature of any pain. All that we can propound concerning it is a relation of the events which seem to lead to it, and the condition of the parts in which it occurs, at the time of its occurrence. We know that the pain is not of that kind which arises from inflammation, or ulceration, or any other fixed physical alteration of a part. All the circumstances attending it prove it to be of that kind which occurs in cramp or spasm, or from pressure, or in the class of cases termed neuralgic, in which the painful sensation is the result of some unknown temporary condition of the nerves of the part, not manifested by any physical alteration of them discoverable by our senses. We have sufficient evidence that such a morbid condition of the nerves may be produced in a heart in all other respects sound; and when it takes place in a heart manifestly diseased in its structure, we must consider the structural lesions merely as predisposing and exciting causes of the pain. That the structural lesions are not the immediate and necessary source of the pain, is sufficiently proved by its intermitting character, and the perfect ease in the intervals, when the structural lesion is precisely the same as during the paroxysm. The anatomical structure, the peculiar action and functions of the heart and annexed great vessels, will sufficiently explain all the modifications of the pain and other phenomena observed in the anginous paroxysm. The radiation of the pain to a distance from the primary and principal site of it is only in conformity with what is observed in all other painful affections: stone in the bladder produces pain in the glans penis: calculus in the ureter, pain in the abdominal walls; inflammation of the cartilages of the hip-joint, pain in the knee; and, what is perhaps a still more appropriate illustration, irritation at the origin of nerves in general frequently manifests itself only by pain at their extremities. In a word, the pain, in the paroxysm of angina, may arise from neuralgia, from spasm, from over-distention; and the other principal phenomena may all, we think, be explained by the derangements of the functions of the heart, considered as a muscular organ charged with the office of circulating the mass of blood." (P. 85.)

"*Anthelmintics*" and "*Antispasmodics*" are concisely and satisfactorily treated upon by Dr. A. Thomson.

The concluding article of the first part of the work, "*Aneurism of the Aorta*," is from the pen of Dr. Hope.

In looking over the various articles of which this first part of the "Cyclopædia of Practical Medicine" consists, there is one apparent departure from the general plan, so well and so attractively laid down in the Prospectus. The subjects which are really practical, and upon which the general practitioner and student are so likely to require information, inasmuch as each day's practice may demand an intimate acquaintance with them, are too briefly considered; while the description of those diseases, (e. g. Acne and Alopecia,) which might more safely have been brief, because they are of less importance and of less frequent occurrence, occupies comparatively too great a space. This is an error which may easily be avoided in the succeeding parts, and we have no doubt it will, as it has been remarked by more than one critical observer, and is indeed too obvious to escape detection.

We look forward to the completion of this work with great interest. In this country its design is novel, and our anxiety for the reputation of English medicine, as well as our good wishes for the Editors and Contributors, induce us to wish most sincerely that its general execution may satisfy the high expectations the Prospectus has excited, and that it may bear comparison with the elaborate productions of a similar kind which, within the last few years, have been published in France and Germany. In such a work we have reason to expect that each subject will be more satisfactorily treated than in a professed compilation by one writer, embracing the whole and vast circle of medical science; for no one man can be equal to the perfect execution of such a task, however great may be his zeal or talent; "sudden fits of inadvertency will surprise vigilance, and casual eclipses of the mind will darken learning." In the Cyclopædia we shall have all the benefits of a division of labour, as it is to be presumed that each subject will be allotted to a "contributor" who has proved that he is capable of treating it with ability. See p. 402.

